# Intestinal helminth infections and associated risk factors among adults in the Lao People’s Democratic Republic

**DOI:** 10.1186/s40249-023-01112-0

**Published:** 2023-06-30

**Authors:** Sengaloun Phonekeo, Sengchanh Kounnavong, Manithong Vonglokham, Latsamy Siengsounthone, Anousin Homsana, Sascha Gummin, Penelope Vounatsu, Prawat Nittiyanant, Suchin Worawichawong, Wichai Aekplakorn, Peter Odermatt, Somphou Sayasone

**Affiliations:** 1 Lao Tropical and Public Health Institute, Ministry of Health, Vientiane, Lao People’s Democratic Republic; 2 Epidemiology and Public Health Department, Swiss Tropical and Public Health Institute, Allschwil, Switzerland; 3 University of Basel, Basel, Switzerland; 4 Ramathibodi Hospital, Faculty of Medicine, Mahidol University, Bangkok, Thailand; 5 Department of Pathology, Ramathibodi Hospital, Faculty of Medicine, Mahidol University, Bangkok, Thailand

**Keywords:** Intestinal helminth, Prevalence, Risk factors, Investigation, Regions, Lao PDR

## Abstract

**Background:**

Helminthiases are highly endemic in Southeast Asia, including the Lao People's Democratic Republic (Lao PDR). This study aimed to assess the current intestinal helminth infections and the associated risk factors among adults across the Lao PDR.

**Methods:**

A cross-sectional survey was conducted in 165 villages across 17 provinces and the Vientiane Capital, Lao PDR. A multi-stage sampling method was employed to select the adult study participants (≥ 18 years). Data collection included (1) interview of the study participants, (2) physical measurements, and (3) a five gram of stool sample from each study participant was collected and preserved in 10% formalin solution for intestinal helminth detection using formalin-ether concentration technique (FECT). Descriptive analysis was used to describe the socio-demographic characteristics of study participants and the prevalence of intestinal helminth infections. Logistic regressions were applied to test the association between intestinal helminth infection and individual risk factors. A *P-value* below 0.05 was considered statistically significant.

**Results:**

A total of 2800 study participants were enrolled. Their average age was 46.0 years; 57.8% were female. Overall, 30.9%, 8.6% and 1.5% of study participants were infected with one, two, or three different intestinal helminth species, respectively. Among the study participants 21.6% were infected with hookworm, 18.8% with *Opisthorchis viverrini-*like (*Ov-*like) infection, 4.8% with *Strongyloides stercoralis*, 2.3% with *Ascaris lumbricoides*, 1.5% with *Trichuris trichiura,* and 3.3% with *Taenia* spp. *Ov-*like infection was of high prevalence in the southern (28.8%) and central (21.3%) provinces, while hookworm (26.3%), *A. lumbricoides* (7.3%), *T. trichiura* (3.1%), and *Taenia* spp. (4.2%) were prevalent in the northern provinces. Risk analysis showed that men were more likely to be infected with hookworm [adjusted odds ratio (a*OR*) = 1.2, *P* = 0.019]. The Lao-Tai ethnic group had a 5.2-times (*P* < 0.001) higher chance of having *Ov-*like infection than the minorities. Possession of toilet facility at home was associated with reduced odds for *Ov-*like (a*OR* = 0.4, *P* < 0.001) and hookworm (a*OR* = 0.6, *P* < 0.001) infections.

**Conclusions:**

Our study provides a nationwide update of the intestinal helminth prevalence among adults in Lao PDR. To the best of our knowledge, this is the first Lao nationwide survey on intestinal helminth infections and risk factors in adults. It provides crucial information for national control programs for intestinal helminth infections in Lao PDR.

**Graphical abstract:**

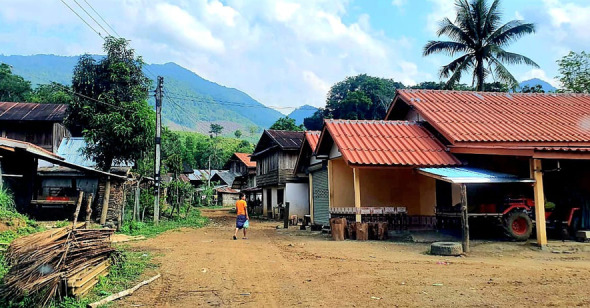

**Supplementary Information:**

The online version contains supplementary material available at 10.1186/s40249-023-01112-0.

## Background

Intestinal helminth infections are a major public health concern in low- and middle-income countries (LMICs). The Lao People’s Democratic Republic (Lao PDR) is one such country in Southeast Asia with distinct eco-epidemiological and socio-economic regions that serve as a niche for particular intestinal helminthiases. The Lao PDR has a north–south dimension of more than 2000 km where mountains and low-land area provide different niches. While the topography in the north is largely mountainous with high humidity, *Ascaris lumbricoides* and *Trichuris trichiura* [1] are endemic*.* Other soil-transmitted helminthiasis (STH) including hookworm and *Strongyloides stercoralis,* are endemic in the entire country [1–4]. The lowlands along the Mekong River are located in the central and southern regions of Lao PDR. There, the small Asian liver fluke, *Opisthorchis viverrini,* is highly endemic [5–8] with prevalence rates exceeding 50% in some places [7–10]. In the most Southern province of Champasack, the eco-epidemiological features of the environment enable the transmission of *Schistosoma mekongi.*

It is well documented that infection with STHs is linked to a variety of morbidities, including malnutrition, growth deficit, and cognitive impairment in children [11–13]. In Lao PDR, the prevalence of malnutrition among pre-school and school-aged children is among the highest in Southeast Asia. In 2017, of the 11,556 children under five years of age, 33% were stunted, 21.1% underweighted, and 9% wasted [14]. Intestinal helminthiases play a significant role in the observed undernutrition prevalence [9]. Furthermore, heavy and chronic infection with *O. viverrini* may cause severe hepatobiliary morbidity, including cholangiocarcinoma, a fatal bile cancer [15–17]. Severe hepato-splenic morbidity is associated to chronic *S. mekongi* infection.

The current World Health Organization (WHO)'s global intestinal helminth control strategy is to reduce morbidity associated with the intestinal helminth infection in at-risk populations with a focus on pre-school and school-aged children [18]. Preventive chemotherapy, i.e. repeated treatment of the at-risk populations combined with information, education and communication campaigns, is the mainstay of the control activities. In Lao PDR, a school deworming program is conducted twice per year (in April and October) in areas where the prevalence of STH is 50% or higher and once per year in the areas where the prevalence of STH is between 20 and 50%. Selected treatment is provided for infected children in the areas where the prevalence is less than 20% [19]. However, pre-school children and adults are not part of this program.

Today, the national helminth control program relies on existing information. However, there is no national survey which may provide an informational overview of intestinal helminth infection and risk factors for the entire country, particularly on the adult population. Therefore, this study aimed to investigate the prevalence of intestinal helminth infections and its associated risk factors among adults aged 18 years and older in Lao PDR.

## Methods

### Study area and population

The Lao PDR is located in Southeast Asia, bordering China in the north, Myanmar in the northwest, Thailand in the west, Vietnam in the east, and Cambodia in the south [20]. It has a total population of about 7.3 million people, according to Lao Statistical yearbook 2021 [20]. Lao PDR spans over a total area of 236,800 km^2^ and consists of 17 provinces and the Capital city. One third of the population (35.7%) lives in urban areas and 49.9% is female. This study focused on the adult population of 18 years and older living in 17 provinces and Vientiane Capital, Lao PDR.

### Field and sampling procedures

This study was a part of the national health survey carried out between March and September 2019 in 17 provinces and the Vientiane Capital, Lao PDR. A multi-stage sampling method was employed to select the national representative villages and participants. First, 165 villages were chosen from the list of 8404 villages across the country available at the Lao Statistic Bureau using a probability proportional to size sampling. Second, a simple random sampling procedure was used to select 20 study households in each selected village. Finally, an adult participant aged 18 years or older in each selected household was selected to participate in the study; if there was more than one adult at home on the survey day, the Kish-grid was applied to randomly select an eligible adult to include in the study.

We used pre-tested questionnaires for data collection, including (1) the World Health Organization questionnaire for surveillance of noncommunicable disease risk factors [21], (2) the asset-based approach recommended by the World Bank for the construction of a household wealth index [22], and (3) additional questions on risk factors for intestinal helminth infections that were developed and added to the questionnaires. Data were collected through face-to-face interviews with study participants. All field data were collected by a trained research team consisting of medical staff from the Lao Tropical and Public Health Institute (Lao TPHI) and the provincial health departments.

In this study, we analyze and report the data pertaining to intestinal helminth infections among the study participants and its underlying risk factors, including demographic characteristics (age, gender, ethnicity, education, and profession), household characteristics (e.g., building materials, electricity, and water supply), asset ownership (e.g., car, motorbike, farm engine, and agricultural land), animal ownership (e.g., buffalo, goat, cow, and pig), personal hygiene (e.g., hand hygiene, wearing shoes, and toilet utility), and raw food consumption (e.g., fish, meat, and pork).

### Laboratory procedures

A morning stool sample of two grams was collected from each study participant. The collected sample was well mixed with 10% formalin solution in a 15 ml tube and transported to the laboratory of the Lao Tropical and Public Health Institute (Lao TPHI), Vientiane Capital, for parasitological analysis. Two weeks after the arrival, the fixed samples were processed and analyzed by experienced microscopists using the Formalin Ether Concentration Technique (FECT) [23]. All intestinal helminth eggs observed under the light microscope were identified, counted, and recorded separately by species. In addition, *O. viverrini* eggs were morphologically differentiated from those of minute intestinal flukes (MIF) by observing the clear shoulders at the operculum as well as by eggshell and knob under a light microscope at high magnification [24, 25]. 10% of reading slides were re-examined by a senior lab technician and any discrepancy in the findings was discussed among lab technicians to obtain the final conclusion.

### Data management and analysis

Data were collected in the field using handheld tablets and a software package of the Census and Survey Processing System (CSPro, United State Census Bureau, USA), which was daily synchronized to the central server of the Lao TPHI. The synchronized data were checked daily by data managers and merged into a single master database after completion of the fieldwork. Downloaded data were double checked for consistency and completeness. This included checking ranges and combinations of variables, detecting and handling missing data, and detecting and handling outliers. The double-checked and completed data were transferred to STATA software version 16.0 (Stata Corporation, College Station, TX, USA) for analysis.

Post-stratification was assigned prior to data analysis for probability weighting account for villages, households, and study individuals. Individual weighting was performed with the inverse of the probability of selection, which was considered as the weight for the individual household. The prefix survey command (svy:) for analysis of weighted data in Stata was used to all data analysis.

Household socio-economic status was assessed using household asset-based approach suggested by World Bank [22, 26]. For this method, the socioeconomic index was built using the principal component analysis (PCA) based on the house construction materials, animal and asset ownership [26]. After the calculation, the study households were divided into five wealth quintiles, with the first quintile representing the poorest and the fifth quintile representing the wealthiest. The age of study participants was classified into five groups: (1) 18–29 years old, (2) 30–44 years old, (3) 45–59 years old, (4) 60–69 years old, and (5) 70 years and older.

Provinces were grouped into three regions. The northern region included seven provinces (Phongsaly, Huaphan, Luangnamtha, Borkeo, Oudomxay, Luangprabang, and Xayabouly); seven provinces in the central region (Xiengkhouang, Xaysomboun, Vientiane, Vientiane Capital, Borikhamxay, Khammouane, and Savannakhet); and four in the southern region (Saravane, Champasack, and Attapeu). According to the Lao census 2015, a study village was classified as located in an urban area if it met at least three of the five following criteria: (1) the village was located in the center of a district or province; (2) more than 70% of households in the village used electricity; (3) more than 70% of households in the village had access to water pipes; (4) the village was accessible by road in both seasons; and (5) the village had a permanent market operating throughout the day [27].

Descriptive statistics were performed by calculating frequency and mean with 95% confidence intervals for categorical and numeric variables, respectively. Bivariate logistic regression models were used to examine the association between intestinal helminth infections and potential risk factors including demographic, socio-economic, personal hygiene, and behavioral data. We used, as previous authors did [28], an association with a significance level below 15% in the bivariate logistic regression model to include in the multiple logistic regression. A *P*-value less than 0.05 was considered statistically significant.

## Results

### Study participants

**Fig. 1:**
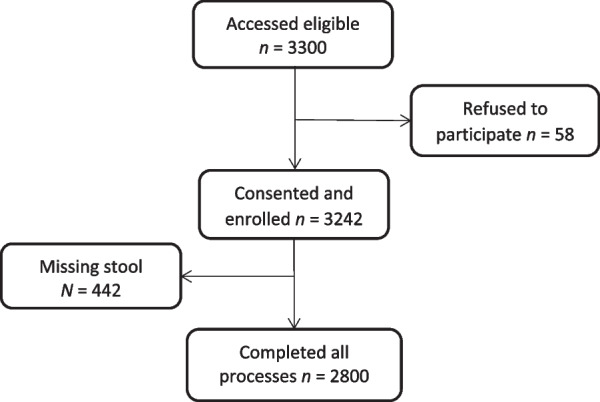
Study diagram

A total of 3300 participants were invited to join the study; 3242 gave their consent among whom and 2800 provided a stool sample for parasitological analysis and were retained for the analysis (Fig. 1).

From the 2800 study participants, 57.8% were women. The average age was 46.0 years [95% confidence interval (*CI*): 45.7–46.2]. About three-quarters of the study participants (76.1%) belonged to the Lao-Tai ethnic group, and 20.2%, 64.3%, and 15.5% were from the northern, central, and southern regions, respectively. About half of study participants (53.1%) lived in urban areas, one-third (31.9%) had no formal education, and 11.5% and 31.3% were classified as belonging to the poorest and richest quintiles, respectively. Overall, men were significantly older than women [47.2 years (46.9–47.5) vs 45.1 years (44.8–45.5)], were significantly more frequently from a minority (28.3% vs 20.6%), attended significantly more frequently University and higher educational levels (16.8% vs 12.5%), were significantly more frequent employees (37.8% vs 31.6%), and were significantly more frequently from the poorest segment (13.8% vs 9.8%) (Table 1).

**Table 1:** Weighted characteristics of adult study participants stratified by gender

Variables	Numbers	Men (*n* = 1254)		Women (*n* = 1546)		Total (*n* = 2800)	
		%	95% *CI*	%	95% *CI*	%	95% *CI*
Age (year)							
Mean age (95% *CI*)	2800	47.2	46.8–47.5	45.1	44.8–45.5	46.0	45.7–46.2
Age group							
18–29	459	11.9	11.1–12.8	16.5	15.6–17.5	14.6	14.0–15.2
30–44	990	36.5	35.1–37.9	35.9	34.7–37.1	36.1	35.2–37.0
45–59	927	31.7	30.5–33.0	35.0	33.8–36.2	33.6	32.8–34.5
60–69	275	12.8	11.8–13.8	8.0	7.4–8.6	10.0	9.5–10.5
≥ 70	149	7.1	6.6–7.7	4.6	4.1–5.1	5.6	5.3–6.0
Ethnicity							
Minority	972	28.3	27.5–29.1	20.6	20.0–21.3	23.9	23.5–24.2
Lao–Tai	1828	71.7	70.9–72.5	79.4	78.7–80.0	76.1	75.8–76.5
Living area							
Urban	986	50.6	49.5–51.7	54.9	54.1–55.7	53.1	53.0–53.2
Rural area	1814	49.4	48.3–50.5	45.1	44.3–45.9	46.9	46.7–46.9
Region							
North	887	20.2	19.6–20.8	20.9	20.4–21.4	20.6	20.5–20.7
Center	1331	64.3	63.4–65.2	61.6	60.9–62.3	62.7	62.6–62.8
South	582	15.5	15.0–16.1	17.5	17.1–17.9	16.7	16.6–16.8
Education level							
University or higher	272	16.8	15.6–18.1	12.5	11.6–13.5	14.3	13.6–15.1
Secondary school	1,242	38.4	37.1–39.6	41.5	40.4–42.6	40.2	39.4–41.0
Primary school	751	36.5	35.1–38.0	26.2	25.1–27.4	30.6	29.7–31.5
Non-formal education	535	8.3	7.8–8.9	19.8	19.0–20.6	14.9	14.4–15.4
Occupation							
Employee	667	37.8	36.4–39.2	31.6	30.4–32.8	34.2	33.4–35.0
Unemployed	211	5.1	4.5–5.7	12.3	11.3–13.3	9.2	8.6–9.9
Farmers	1,527	40.4	39.3–41.5	43.5	42.6–44.4	42.2	41.7–42.7
Retired	395	16.7	15.8–17.6	12.6	11.8–13.4	14.4	13.8–14.9
Wealth index							
Richest	552	28.7	27.4–30.2	33.2	32.1–34.4	31.3	32.1–34.4
Least poor	577	22.5	21.3–23.7	25.5	24.5–26.5	24.2	23.5–25.0
Poor	578	18.1	17.2–19.0	18.9	18.1–19.7	18.5	18.0–19.1
Poorer	552	16.9	16.1–17.6	12.6	12.0–13.2	14.4	14.0–14.8
Poorest	541	13.8	13.1–14.5	9.8	9.3–10.3	11.5	11.2–11.8

### Intestinal helminth infections

Among the 2800 study adults, 30.9% had at least one intestinal helminth infection, 8.6% had two intestinal helminth species, 1.5% had three intestinal helminth species and 59.0% were free from intestinal helminth infections. Hookworm and *Opisthorchis viverrini-*like (*Ov-*like) infections were diagnosed in 21.6% and 18.8% of study participants, respectively. Other intestinal helminths species were much less prevalent, i.e., *S. stercoralis* in 4.8%, *Taenia* spp*.* in 3.3%, *A. lumbricoides* in 2.3%, and *T. trichiura* in 1.5%.

**Fig. 2:**
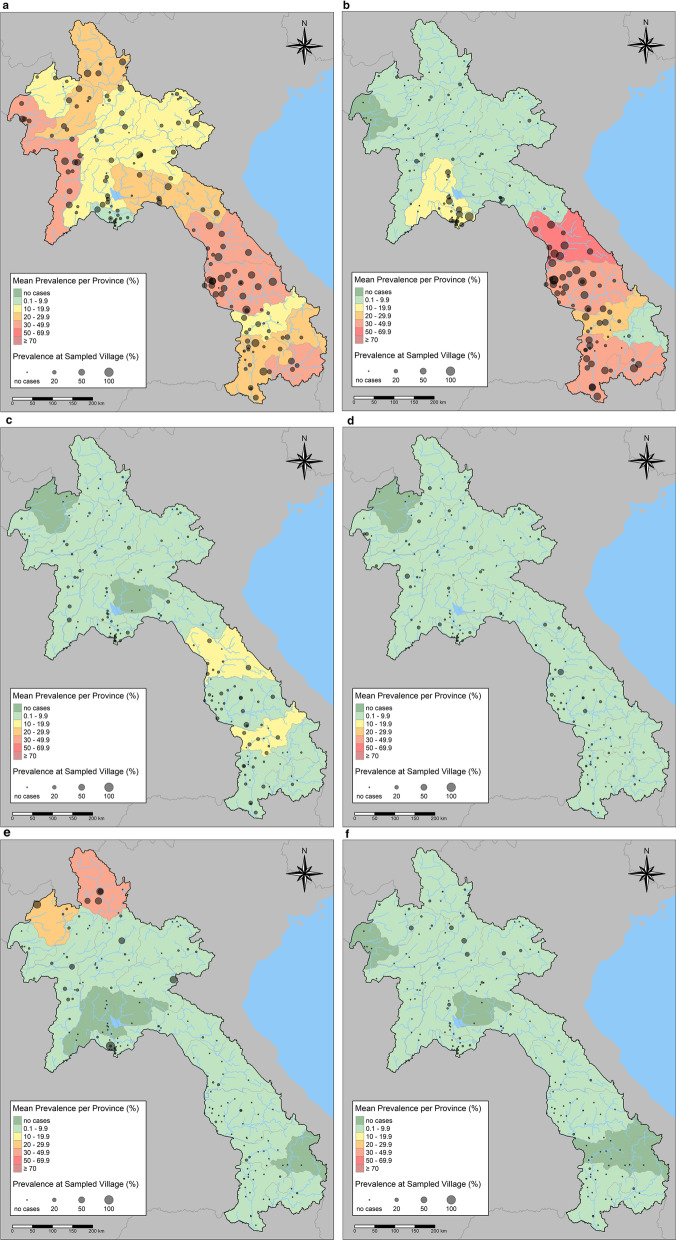
Intestinal helminth infection prevalence map of Lao PDR by enrolled villages and provinces for hookworm (**a**), *Opisthorchis viverrini*-like (**b**), *Strongyloides stercoralis* (**c**), *Taenia* spp. (**d**), *Ascaris lumbricoides* (**e**), and *Trichuris trichiura* (**f**)

The intestinal helminth infections were geographically distributed for each species (Fig. 2). Hookworm infection prevalence was highest in northwest, central, and most southern provinces (Fig. 2a). High *Ov-*like prevalence was observed in central and southern Lao PDR (Fig. 2b). *S. stercoralis* was more prevalent in central and southern provinces (Fig. 2c), *Taenia* spp. infections (Fig. 2d) were endemic in all provinces, *A. lumbricoides* was highest in the most northern provinces (Fig. 2e) and *T. trichiura* prevalence was high in some norther Lao villages but had had low prevalence in all provinces (Fig. 2f). More details of intestinal helminth infections by province are provided in Additional file 1: Table S1.

Furthermore, hookworm was significantly more prevalent in males (23.2%, 95% *CI*: 22.1–24.2) than females (20.4%, 95% *CI*: 19.5–21.3), with the highest prevalence occurring in early adulthood (18–29 years) and among minorities (31.3%, 95% *CI*: 30.2–32.4). *Ov-*like infection rates were similar for males (18.9%) and females (18.7%). The southern region (28.8%), participants aged 45–59 years (21.5%), and Lao-Tai group (21.8%) had the highest rates for *Ov-*like infection. Other less common intestinal helminths, i.e., *S. stercoralis* (6.1% vs 3.6%), *Taenia* spp. (3.8% vs 2.9%), *A. lumbricoides* (3.3% vs 1.3%), and *T. trichiura* (1.8% vs 1.2%) were more prevalent in minorities than the Lao-Tai ethnic group. For all intestinal helminth species, study participants who were from rural areas had a higher infection rate than those from urban areas (Table 2).

**Table 2:** Weighted prevalence of intestinal helminth infections detected in stool samples using formalin-ethyl acetate concentration technique among Lao adults stratified by gender, age group, living area, and region

Variables	Numbers	Hookworm		*Opisthorchis viverrini-*like infection		*Strongyloides stercoralis*		*Taenia* spp*.*		*Ascaris lumbricoides*		*Trichuris trichiura*	
		Prevalence (%)	95% *CI*	Prevalence (%)	95% *CI*	Prevalence (%)	95% *CI*	Prevalence (%)	95% *CI*	Prevalence (%)	95% *CI*	Prevalence (%)	95% *CI*
Total	2800	21.6	20.9–22.2	18.8	18.2–19.4	4.8	4.4–5.1	3.3	3.1–3.6	2.3	2.1–2.4	1.5	1.3–1.7
Sex													
Female	1546	20.4	19.5–21.3	18.7	17.8–19.6	3.0	2.7–3.4	2.3	2.0–2.6	2.7	2.4–2.9	1.3	1.3–1.7
Male	1254	23.2	22.1–24.2	18.9	17.9–19.9	7.1	6.5–7.8	4.8	4.3–5.3	1.8	1.5–2.0	1.7	1.4–1.6
Age group													
18–29	459	25.6	23.6–27.8	16.8	14.8–18.9	3.4	2.6–4.3	3.8	3.0–4.8	4.8	4.2–5.6	2.1	1.2–3.5
30–44	990	23.3	22.1–24.5	17.3	16.2–18.4	3.4	3.0–3.8	3.5	3.1–4.0	2.3	2.0–2.7	1.5	1.3–1.8
45–59	927	18.2	17.2–19.2	21.5	20.1–22.3	6.0	5.4–6.7	3.0	2.6–3.5	1.3	1.2–1.5	1.3	1.1–1.7
60–69	275	23.0	20.7–25.6	17.6	15.7–19.7	8.5	6.9–10.5	2.4	1.5–3.8	2.6	2.4–2.8	0.9	0.7–1.3
70 +	149	17.3	15.6–19.1	21.2	19.0–24.4	3.4	2.6–4.3	4.5	3.8–5.4	0.5	0.3–0.7	1.3	0.7–2.4
Ethnicity													
Lao-Tai	1,828	18.5	17.8–19.3	21.8	21.1–22.6	3.8	3.3–4.3	3.1	2.8–3.5	1.1	1.0–1.3	0.7	0.5–1.0
Minorities	972	31.3	30.2–32.4	9.0	8.3–9.7	5.1	4.7–5.5	4.0	3.5–4.5	5.9	5.5–6.4	3.9	3.6–4.2
Living area													
Urban	986	16.0	15.1–16.9	13.2	12.3–14.1	3.6	3.1–4.1	2.9	2.5–3.4	1.3	1.1–1.6	1.2	0.9–1.5
Rural	1814	27.9	27.1–28.7	25.1	24.3–25.9	6.1	5.6–6.6	3.8	3.5–4.2	3.3	3.1–3.6	1.8	1.6–2.0
Region													
North	887	26.3	25.4–27.3	3.1	2.7–3.6	3.4	3.1–3.9	4.2	3.7–4.7	7.3	6.8–7.9	3.1	2.8–3.4
Central	1331	20.3	19.4–21.2	21.3	20.4–22.2	4.5	4.0–5.0	3.2	2.8–3.6	0.9	0.7–1.1	1.2	0.9–1.6
South	582	20.5	19.5–21.5	28.8	27.7–29.8	4.8	4.4–5.1	2.8	2.4–3.3	1.3	1.2–1.4	0.5	0.3–0.9

*CI* confidence interval

### Risk factors associated with intestinal helminth infections

The weighted logistic regressions associated between two intestinal helminth infections of public health concern (hookworm and *Ov*-like) and predictive risk factors are summarized in Table 3. For other less common intestinal helminth infections, e.g., *S. stercoralis, Taenia* app., *A. lumbricoides,* and *T. trichiura*, the analysis is provided in Additional file 2: Table S2. Overall, the analysis showed that males had a significantly higher chance of hookworm infection than females [adjusted odds ratio (a*OR*) = 1.2, *P* = 0.019]. The Lao-Tai ethnic group had a strongly increased risk for *Ov-*like infection (a*OR* = 4.3, *P* < 0.001), while the risk of hookworm infection was significantly reduced among minorities (a*OR* = 0.5, *P* < 0.001). Participants who were classified as the poorest group had the highest risk for both hookworm (a*OR* = 4.1, *P* < 0.001) and *Ov-*like (a*OR* = 2.7, *P* < 0.001) infections when compared to the richest one. Participants who were living in a rural area had 1.2 times (*P* < 0.001) and 2.0 times (*P* < 0.001) higher risk of hookworm and *Ov-*like infections. Participants who were living in the central (a*OR* = 8.1, *P* < 0.001) and southern (a*OR* = 7.8, *P* < 0.001) provinces were more likely to have *Ov-*like infections than those living in the northern provinces. Participants who reported having a latrine facility at home had a reduced risk for both hookworm (a*OR* = 0.4, *P* < 0.001) and *Ov-*like (a*OR* = 0.4, *P* < 0.001) infections. Reported consumption of raw or undercooked freshwater fish in the last seven days was significantly associated with *Ov-*like infection (a*OR* = 1.3, *P* < 0.001) compared to those who did not consume the raw or undercooked fish in the same period.

**Table 3:** Weighted (SVY) logistic regression models associated between two major intestinal helminth infections (hookworm and *Ov-*like) and demographic, socio-economic, personal hygiene and behavioral data among adult participants

Variables	Hookworm				*Ov*-like infection			
	c*OR* (95% *CI*)	*P*-value	a*OR* (95% *CI*)	*P*-value	c*OR* (95% *CI*)	*P*-value	a*OR* (95% *CI*)	*P*-value
Gender								
Female	1.0		1.0		1.0		*	
Male	1.2 (1.1–1.4)	0.019	1.2 (1.1–1.4)	0.019	1.1 (0.9–1.3)	0.402		
Age group								
18–29	1.0				1.0			
30–44	0.8 (0.7–0.9)	0.033	**		1.0 (0.9–1.2)	0.670	*	
45–59	0.6 (0.5–0.8)	< 0.001	**		1.3 (1.1–1.6)	0.001	**	
60–69	0.7 (0.6–0.9)	0.001	**		1.1 (0.9–1.3)	0.551	*	
70 +	0.6 (0.5–0.7)	< 0.001	**		1.4 (1.1–1.7)	0.005	**	
Ethnic groups								
Minorities	1.0		1.0		1.0		1.0	
Lao-Tai	0.5 (0.4–0.6)	< 0.001	0.7 (0.6–0.8)	< 0.001	2.8 (2.6–3.1)	< 0.001	4.3 (3.8–4.8)	< 0.001
Education level								
No formal education	1.0		1.0		1.0		1.0	
Primary school	0.8 (0.7–0.9)	< 0.001	1.6 (1.4–1.8)	< 0.001	0.7 (0.6–0.8)	< 0.001	0.7 (0.6–0.8)	< 0.001
Secondary school	1.1 (1.0–1.2)	0.019	1.6 (1.4–1.7)	< 0.001	1.2 (1.1–1.3)	0.025	**	
University	0.6 (0.5–0.7)	< 0.001	1.7 (1.4–2.1)	< 0.001	0.5 (0.4–0.6)	< 0.001	0.5 (0.4–0.7)	< 0.001
Occupation								
Employees	1.0		1.0		1.0		1.0	
Students	1.1 (0.9–1.4)	0.237	*		0.8 (0.6–1.1)	0.075	0.7 (0.6–0.9)	0.023
Farmers	1.9 (1.7–2.1)	< 0.001	**		1.8 (1.6–2.0)	< 0.001	1.3 (1.2–1.5)	< 0.001
Retired/elderly	1.2 (1.1–1.4)	0.003	0.6 (0.4–0.8)	0.002	1.4 (1.2–1.6)	< 0.001	**	
Wealth index								
Richest	1.0		1.0		1.0		1.0	
Least poor	2.0 (1.7–2.4)	< 0.001	2.0 (1.6–2.4)	< 0.001	1.7 (1.4–1.9)	< 0.001	1.2 (1.1–1.4)	0.029
Poor	3.3 (2.9–3.9)	< 0.001	3.1 (2.6–3.9)	< 0.001	2.3 (1.9–2.6)	< 0.001	1.4 (1.2–1.7)	< 0.001
Poorer	3.0 (2.6–3.5)	< 0.001	2.6 (2.2–3.1)	< 0.001	2.2 (1.9–2.6)	< 0.001	1.4 (1.1–1.6)	0.002
Poorest	4.1 (3.5–4.7)	< 0.001	3.2 (2.6–3.8)	< 0.001	2.7 (2.3–3.2)	< 0.001	1.9 (1.6–2.4)	< 0.001
Residential area								
Urban	1.0		1.0		1.0		1.0	
Rural	2.0 (1.9–2.2)	< 0.001	1.2 (1.1–1.4)	< 0.001	2.2 (2.0–2.4)	< 0.001	2.0 (1.8–2.2)	< 0.001
Regions								
Northern	1.0		1.0		1.0		1.0	
Central	0.7 (0.6–0.8)	< 0.001	**		8.5 (7.2–10.0)	< 0.001	8.1 (6.8–9.7)	< 0.001
Southern	0.7 (0.6–0.8)	< 0.001	0.7 (0.6–0.8)	< 0.001	12.7 (10.8–14.9)	< 0.001	7.8 (6.5–9.3)	< 0.001
Having toilet at home								
No	1.0		1.0		1.0		1.0	
Yes	0.8 (0.7–0.9)	< 0.001	0.6 (0.5–0.7)	< 0.001	1.7 (1.4–2.1)	< 0.001	0.4 (0.3–0.5)	< 0.001
Eating undercooked/raw fish in last 7 days								
No	1.0		**		1.0		1.0	
Yes	1.2 (1.1–1.3)	0.001			1.5 (1.4–1.7)	< 0.001	1.3 (1.2–1.5)	< 0.001
Eating raw meat (cow, buff, goat) in last 7 days								
No	1.0		**		1.0		1.0	
Yes	1.2 (1.1–1.4)	0.001			1.2 (1.1–1.4)	< 0.001	1.4 (1.2–1.6)	< 0.001

c*OR*: crude Odds Ratio obtained from bi-variable analysis; a*OR*: adjusted Odds Ratio obtained from multivariate analysis; *CI*: Confidence interval; *SVY*: Survey command

*Not included in model, *P* > 0.15; **Not Significant, *P* > 0.05

## Discussion

In Lao PDR, helminth species such as *O. viverrini*, STH, and *Taenia* spp. are endemic [1, 9]. In this study, we report on intestinal helminth infections in a nationally representative sample of adults enrolled in the national health survey conducted in 2019, and almost half (44.1%) of the participants were detected with intestinal helminth eggs in their stool samples. To our knowledge, this is the first national study conducted among adults to assess the intestinal helminth infections in the country.

Among our study participants, hookworm infection had the highest prevalence at 21.6%. This finding is similar to the report from previous studies, which found the prevalence of hookworm between 19.1% and 27.8% in different provinces [29, 30]. Although there has been no deworming program for adults until today, it is thought that the intervention in school-aged children (the school deworming program), which has been actively implemented across the country [19], coupled with significant economic growth over the past decades, should lead to a reduction of STH infections, including hookworm, in adults. The observed high hookworm prevalence in this study could be multi-factorial: (1) high exposure, (2) low efficacy, and (3) variable transmissibility among hookworm species. Regarding (1) high exposure, the majority of Lao people are intensively engaged in subsistence rice farming, a practice that increases hookworm exposure and transmission. Per (2) low efficacy, it has been shown that orally administered single-dose mebendazole (500 mg), which is used as the drug of choice in school deworming programs and is widely available at local health facilities for treatment of STH infections, has low efficacy against hookworm in Lao PDR, particularly as a single dose [31, 32]. Finally, concerning (3) variable transmission among hookworm species, it might be possible that some hookworm infections are caused by animal origins such as *Ancylostoma ceylanicum,* which is frequently reported in the region and neighboring countries [33]. Albendazole and mebendazole were shown to have low efficacy against *A. ceylanicum* hookworm [34].

Our study found an *Ov*-like infection in 18.8% of the study adults. The prevalence was high in the southern (28.8%) and central (21.3%) provinces, while in the northern provinces, the prevalence was only 3.1%. Interestingly, our study showed a significantly lower prevalence of *Ov-*like infection than a previous study conducted in nine provinces in Lao PDR, which reported a prevalence of *Ov-*like infection of 55.6% among the study participants [29]. It is important to note that the previous study reported findings from a series of studies conducted between 2007 and 2011 using the Kato-Katz technique as a tool for parasitological diagnosis, while our study was conducted in 2019 and used the FECT method for diagnosis, which might yield different helminth prevalence. In addition, our study demonstrated an almost two-fold higher prevalence than that of a national study conducted in 2002 among school-aged children, which found the prevalence of *Ov-*like at only 10.9% [30]. The difference in observing the *Ov-*like infection in the later two studies may be due to the fact that *Ov*-like infection depends on the frequency of raw or undercooked fish consumption. Adults actively participate in social events where the raw/undercooked fish dishes are commonly prepared and shared through traditional dishes, thus exposing them to higher risk of *Ov-*like infection than children [35].

Our data analysis showed that males were significantly more infected with hookworm than women. This finding is in line with other previous studies conducted in Lao PDR [36] and neighboring Thailand [37, 38]. Traditionally, men are more engaged in outdoor activities, e.g., working in rice fields, which exposes them to a greater risk of infection. The *Ov-*like infection had a high prevalence in the central and southern provinces, and among members of the Lao-Tai ethnic group were more likely infected. This observation is also in line with previous studies, which reported the high prevalence of *Ov-*like infections in the central and southern provinces and that the Lao-Tai ethnicity was more susceptible to the infection [29]. The majority of the Lao-Tai ethnic group lives in the lowland area along the Mekong River, where different types of *Cyprinoid* fish are abundant. Two traditional dishes, e.g., Lappa (chopped fish and mixed ingredients, chili, and herbs) and Koipa (minced fish, mixed with ingredients, chili, and herbs), expose the Lao-Tai ethnic group to the risk of *Ov-*like infection, as these dishes are mostly served raw and preferentially consumed during social events [39]. Participants who were classified in the poorest quintile had the highest risk for hookworm (a*OR* = 3.2, *P* < 0.001) and *Ov-*like (a*OR* = 1.9, *P* < 0.001) infections. These observations are similar to the findings from studies conducted in Malaysia and the Philippines, which showed the low family incomes [40–42] were significantly associated with intestinal helminth infections. Other studies conducted in Thailand and Vietnam concluded that socio-economic disadvantage is a predictive factor for *Ov-*like infection [43–45]. Study participants who had a latrine at home exhibited a significantly reduced risk for hookworm (a*OR* = 0.06, *P* < 0.001) and *Ov-*like infections (a*OR* = 0.4, *P* < 0.001). This finding is in line with the results of a previous study conducted in northern Lao PDR, which concluded that the presence of a latrine at home decreased the risk of hookworm infection (a*OR* = 0.278, *P* = 0.006) [36]. Another study conducted in the Philippines also reported that defecating openly was significantly associated with STH infections [40].

The main limitation of our study is the relatively low sensitivity of the FECT method on a single stool sample, particularly for the detection of *S. stercoralis* and *Taenia* spp. infection in the preserved stool samples. The low prevalence of *S. stercoralis* and *Taenia* spp. observed in this study is most likely due to the fact that FECT analysis is unable to detect these infections [46, 47]. The use of more sensitive diagnostic techniques, e.g., the Baermann test or agar plate culture for *S. stercoralis* [48] or a coproantigen ELISA for *Taenia* spp. infection [49, 50], could provide a considerably higher prevalence.

In this study, we collected a preserved stool sample from each study participant and analyze it using FECT in order not to hamper or delay the overall activities of the national health survey, which covered an array of field data collection. Therefore, we likely underestimate the true prevalence of all intestinal helminth species. In addition, FECT did not allow for the assessment of infection intensity, which is a very useful indicator of morbidity. Furthermore, examining multiple fecal samples per person would also result in a higher yield in egg discovery [51]. Lastly, the eggs of *O. viverrini* and minute intestinal flukes (MIF) are very similar in shape and size. Previous studies have demonstrated that MIF, i.e., *Haplorchis* sp. and others, are endemic in several provinces of Lao PDR. Morphological identification under a light microscope during the FECT analysis might confuse the identification between MIF and *O. viverrini* eggs. Hence, we reported this study as an *Ov*-like infection to avoid overestimating the prevalence of *O. viverrini* in some endemic areas.

## Conclusions

Helminthiases remain a significant public health problem in Lao PDR. Our study demonstrates that intestinal helminthiases are highly endemic nationwide and represent a public health concern. *Ov-*like infection is highly endemic in the southern and central provinces, while hookworm is a common STH prevalent across the country, especially in the north. Personal hygiene and behaviors, e.g., consuming raw fish and meat through traditional dishes, are identified as predictive factors associated with intestinal helminthiases. Therefore, a health education campaign on the consumption of cooked food and personal hygiene practices in the endemic communities might be an effective control program. Furthermore, our study shows that study participants who are from households with sanitary facilities have a lower risk for helminth infections. Hence, promotion the use of sanitary facilities to reach the nationwide goal to be open-defecation free will significantly reduce the prevalence of helminths in Lao PDR.

## Supplementary Information

**Additional file 1.** Weighted prevalence of intestinal helminth infections among adults enrolled in the study, stratified by province.

**Additional file 2.** Weightedlogistic regression models associated between less common intestinal helminth infections and demographic, socio-economic, personal hygiene, and behavioral data among adult participants.

## Data Availability

Data is available at the Lao TPHI and collaborators’ institutions and fully accessible to all co-authors. Data can be shared with other institutions and researchers upon reasonable request.

## Abbreviations

Lao PDR: Lao People’s Democratic Republic
a*OR*: Adjusted *odds ratio*
*CI*: Confidence interval
c*OR*: Crude odds ratio
*O. viverrini*: *Opisthorchis viverrini*
*Ov*-like: *Opisthorchis viverrini-*Like
EPG: Eggs per gram
STH: Soil-transmitted helminths
WHO: World Health Organization
MDA: Mass drug administration
FECT: Formalin-ethyl acetate concentration technique
CSPRO: Census and Survey Processing System
SVY: Survey command
PCA: Principal component analysis
*SD*: Standard deviation
