# Supplementary material for: Intestinal helminth infections and associated risk factors among adults in the Lao People’s Democratic Republic
Source: Infect Dis Poverty. 2023 Jun 30;12:61. doi: 10.1186/s40249-023-01112-0 (PMC10311807; doi:10.1186/s40249-023-01112-0)
Supplement: Supplementary file 1 — Additional file 1. Weighted prevalence of intestinal helminth infections among adults enrolled in the study, stratified by province. [file 40249_2023_1112_MOESM1_ESM.docx]

| **Suppl. 1: Weighted prevalence of intestinal helminth infections among adults enrolled in the study, stratified by province** | | | | | | | | | | | | | | |
| --- | --- | --- | --- | --- | --- | --- | --- | --- | --- | --- | --- | --- | --- | --- |
| **Province** | **No. of tests** | **Hookworm** | | ***Ov*-like infection** | | ***S. stercoralis*** | | ***Taenia* spp.** | | ***A. lumbricoides*** | | ***T. trichiura*** | |  |
|  |  | **%** | **95% CI** | **%** | **95% CI** | **%** | **95% CI** | **%** | **95% CI** | **%** | **95% CI** | **%** | **95% CI** |  |
| Vientiane Capital | 342 | 5.6 | 4.7-6.8 | 16.7 | 15.1-18.5 | 3.4 | 2.6-4.4 | 2.3 | 1.6-3.2 | 1.0 | 0.6-1.5 | 0.7 | 0.5-1.0 |  |
| Savannakhet | 315 | 42.6 | 39.3-46.0 | 40.3 | 37.7-42.9 | 7.1 | 5.8,8.7 | 3.6 | 2.7-4.8 | 0.3 | 0.1-1.0 | 1.5 | 0.6-3.5 |  |
| Champasack | 292 | 18.6 | 17.7-19.5 | 30.3 | 28.9-31.1 | 7.3 | 6.6-8.1 | 2.1 | 1.6-2.7 | 0.3 | 0.1-0.5) | 0.5 | 0.3-0.7 |  |
| Luangprabang | 204 | 25.9 | 24.5-27.4 | 2.9 | (2.1-4.0 | 1.3 | 0.7-2.4 | 1.8 | 1.2-2.8 | 6.8 | 6.1-7.5 | 7.7 | 7.1-8.5 |  |
| Vientiane province | 203 | 16.7 | 15.2-18.4 | 15.9 | 14.2-17.8 | 4.0 | 3.1-5.1 | 2.3 | 1.8-2.9 | 0 | 0 | 2.5 | 2.3-2.8 |  |
| Xayabuly | 189 | 33.6 | 31.8-35.6 | 4.6 | 4.0-5.2 | 8.7 | 7.6-9.9 | 5.8 | 5.1-6.6 | 9.5 | 8.2-11.0 | 0.3 | 0.2-0.4 |  |
| Saravane | 172 | 15.8 | 14.1-17.7 | 24.5 | 22.2-26.9 | 10.6 | 9.3-12.1 | 3.8 | 3.1-4.8 | 3.4 | 3.1-3.9 | 0 | 0 |  |
| Khammouane | 170 | 35.5 | 31.5-39.8 | 43.1 | 39.4-46.9 | 9.7 | 8.1-11.5 | 3.9 | 3.0-5.2 | 0.7 | 0.5-0.8 | 2.8 | 1.4-5.5 |  |
| Borikhamxay | 159 | 23.3 | 21.6-25.0 | 8.9 | 7.9-9.9 | 3.1 | 2.5-3.9 | 5.6 | 5.2-6.1 | 2.3 | 2.1-2.5 | 0.9 | 0.6-1.4 |  |
| Oudomxay | 146 | 22.9 | 20.7-25.2 | 3.5 | 2.5-5.5 | 3.4 | 2.5-4.5 | 4.4 | 3.5-5.4 | 3.1 | 2.4-4.0 | 2.6 | 2.2-3.1 |  |
| Huaphanh | 130 | 18.2 | 16.5-20.1 | 1.2 | 0.9-1.5 | 2.6 | 2.3-2.9 | 3.4 | 2.7-4.2 | 1.4 | (0.8-2.4 | 1.7 | 1.4-2.1 |  |
| Xiengkhuang | 104 | 15.1 | 13.5-16.8 | 1.1 | 0.6-2.1 | 1.3 | 0.8-2.3 | 3.1 | 2.2-4.5 | 1.6 | 1.3-2.0 | 0 | 0 |  |
| Phongsaly | 82 | 29.5 | 24.5-35.1 | 3.0 | 1.2-7.1 | 0.3 | 0.2-0.4 | 7.3 | 4.3-12.0 | 22.4 | 19.8-25.2 | 0.7 | 0.3-1.5 |  |
| Borkeo | 72 | 31.0 | 27.4-34.9 | 0 | 0 | 1.4 | 1.4-1.4 | 8.1 | 5.8-11.2 | 2.0 | 1.2-3.5 | 0 | 0 |  |
| Attapeu | 66 | 36.6 | 32.0-41.5 | 39.9 | 36.3-43.5 | 3.0 | 1.9-4.6 | 3.7 | 2.2-6.2 | 0.1 | <0.1-0.2 | 2.2 | 1.0-4.7 |  |
| Luangnamtha | 64 | 10.6 | 7.3-15.2 | 2.6 | 1.7-4.1 | 0 | 0 | 0 | 0 | 14.2 | 11.1-17.9 | 3.4 | 1.6-7.5 |  |
| Sekong | 52 | 25.8 | 19.3-33.6 | 7.4 | 3.7-14.2 | 1.0 | 0.8-1.1.2 | 1.0 | 0.8-1.1.2 | 0 | 0 | 0 | 0 |  |
| Xaysomboun | 38 | 23.3 | 21.9-24.7 | 2.2 | 1.2-4.1 | 0 | 0 | 3.0 | 3.0-3.0 | 0 | 0 | 0 | 0 |  |
